# Delayed development of the protective IL-17A response following a *Giardia muris* infection in neonatal mice

**DOI:** 10.1038/s41598-019-45544-x

**Published:** 2019-06-20

**Authors:** Oonagh Paerewijck, Brecht Maertens, Aurélie Gagnaire, Karolien De Bosscher, Peter Geldhof

**Affiliations:** 1 Department of Virology, Parasitology and Immunology, Laboratory of Parasitology, Faculty of Veterinary Medicine, Ghent University, Merelbeke, Belgium; 2 VIB Department of Medical Protein Research, Receptor Research laboratories, Nuclear Receptor Lab, Faculty of Medicine and Health Sciences, Ghent University, Ghent, Belgium

**Keywords:** Antimicrobial responses, Parasite host response

## Abstract

*Giardia* is an intestinal protozoan parasite that has the ability to infect a wide range of hosts, which can result in the clinical condition ‘giardiasis’. Over the years, experimental research has shown the crucial involvement of IL-17A to steer the protective immune response against *Giardia*. The development of the protective response, as reflected by a significant drop in cyst secretion, typically takes around 3 to 4 weeks. However, early-life infections often have a more chronic character lasting for several weeks or months. Therefore, the aim of the current study was to investigate the dynamics of a *Giardia muris* infection and the subsequent host immune response in neonatal mice infected 4 days after birth. The outcome of the study showed that a *G*. *muris* infection in pre-weaned mice failed to trigger a protective IL-17A response, which could explain the prolonged course of infection in comparison to older mice. Only after weaning, a protective intestinal immune response started to develop, characterized by an upregulation of IL-17A and Mbl2 and the secretion of parasite-specific IgA.

## Introduction

*Giardia* is a highly prevalent intestinal protozoan parasite with a wide vertebrate host range, including humans. An infection with *Giardia* can either pass asymptomatically or can in some cases lead to the clinical condition of giardiasis, which is characterized by gastro-intestinal complaints such as diarrhoea, abdominal pain, nausea and weight loss^1,2^.

In recent years, it has become increasingly evident that the development of protective immunity against a *Giardia* infection is regulated by the IL-17A/IL-17RA axis^3^. Several studies reported the upregulation of IL-17A during infections in both mice and cattle^4–6^. Furthermore, re-stimulation of CD4+ T cells from human patients that were previously infected with *Giardia* resulted in an upregulation of IL-17^7^. IL-17A subsequently triggers the production of a range of antimicrobial peptides and complement factors and regulates the secretion of parasite-specific IgA’s in the intestine^5,8^. The binding of these components to the *Giardia* trophozoites is thought to be crucial to clear the infection from the intestine. Infection studies, performed in mice, gerbils, cats and dogs^4,9,10^, show that the development of immunity, as reflected by a significant drop in cyst secretion, typically takes around 3 to 4 weeks. In contrast, other studies describe chronic *Giardia* infections that last for several weeks to even months in calves^6^, goats^11^ and sheep^12^. Importantly, chronic infections are typically observed following early-life infections in the first days or weeks after birth. For instance in cattle, the prevalence is often higher in pre-weaned calves, with a peak prevalence between 1 and 8 weeks of age^13,14^, than in weaned calves^15,16^. Likewise, a high *Giardia* prevalence in young animals is also common in for instance reindeers^17^, lambs^18^, cats and dogs^19^. In humans, *Giardia* is also more prevalent in children, with a peak prevalence in infants and pre-school children, and is declining with increasing age^20^.

These observations suggest that there might be an age dependent effect on the immune development process against this parasite. The aim of the current study was to investigate the dynamics of a *G*. *muris* infection and the subsequent host immune response in neonatal mice infected 4 days after birth.

## Materials and Methods

### Infection experiments and tissue sample collection

All animal experiments were conducted in accordance with the E.U. Animal Welfare Directives and VICH Guidelines for Good Clinical Practice. Ethical approval to conduct the studies was obtained from the Ethical Committee of the Faculty of Veterinary Medicine, Ghent University (approval number EC 2017/97).

**Figure 1:**
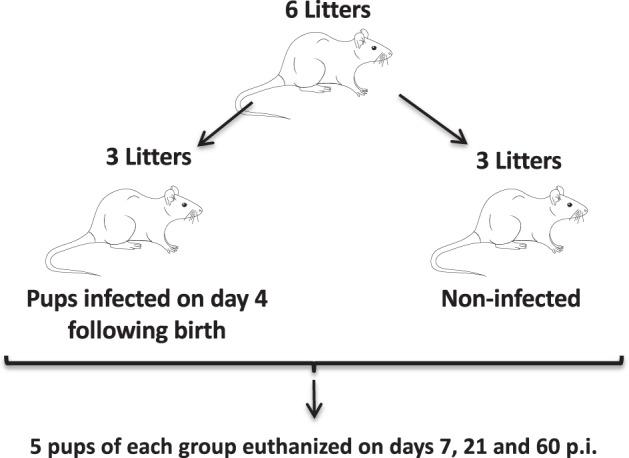
Study design of the artificial infection experiment in neonatal mice. Male and female pups from 3 litters were infected 4 days after birth with *G*. *muris* cysts. At days 7, 21 and 60 post-infection, corresponding with days 11, 25 and 64 after birth, respectively, 5 pups were selected at random from both the infected and control litters. The pups were euthanized, tissue samples were collected and trophozoites present in the small intestine counted. Faecal cyst counts were performed every 2 days from day 21 p.i. onwards.

The setup of the artificial infection study is schematically shown in Fig. 1. C57Bl/6 pups, both males and females, were collected from 6 different litters. The pups from 3 litters were infected 4 days after birth with 10*3 *G*. *muris* cysts suspended in 20 μL phosphate-buffered saline (PBS), administered orally with a pipet. The pups from the other litters remained uninfected and received only PBS and thus served as controls. All pups were weaned at 28 days old. At day 7, day 21 and day 60 post-infection, 5 pups were randomly selected from both the infected and control litters. At the time of necropsy, a 2 cm long duodenal tissue sample was taken from each animal. The samples were snap-frozen in liquid nitrogen and subsequently used for the extraction of total RNA. In short, samples were crushed, resuspended in TRIzol (Invitrogen) and RNA was further purified using the RNeasy minikit (Qiagen), according to the manufacturer’s instructions. This protocol was complemented with the RNase-free DNase set (Qiagen) for the removal of contaminating genomic DNA by on-column DNAse digestion. The quality of the isolated RNA was verified with an Experion automated electrophoresis system (Bio-Rad) and the concentration was measured with a NanoDrop ND-1000 spectrophotometer (NanoDrop Technologies).

From day 21 p.i. onwards, cyst excretion was monitored every 2 days as previously described by Dreesen *et al*.^4^. At the time of necropsy, the number of trophozoites present in the intestine was counted as previously described by Paerewijck *et al*.^8^ In short, the small intestine was removed, starting from 2 cm behind the first part of the duodenum. The segment was placed in PBS and incubated on ice for 20 minutes before counting the trophozoites by using a hemacytometer. Results are expressed as the absolute number of trophozoites present in the small intestine.

### Real-time quantitative PCR

The relative mRNA expression levels of several genes in the small intestine of uninfected and infected mice were measured at the different time points by a quantitative Real-Time PCR (qRT-PCR) approach. This was done using the SYBR green master mix (Applied Biosystems) in combination with a StepOnePlus real-time PCR system (Applied Biosystems). The sequences of all primers used are summarized in Table 1. QRT-PCR and normalization of the data, which was based on the housekeeping genes Hprt1, Tbp and Rplpo, was essentially performed as previously described^4^. Gene transcription levels were evaluated based on fold difference between the different groups.

**Table 1:** List of primer sequences used for qPCR analysis of immune-related genes.

Gene symbol	Accession number	Primer sequence
IL17A	NM_010552	F: TGACAGTGGTTTATGCAGAGAC
		R: CGTCACGTCCATCTTTGCC
MBL2	NM_010776	F: ACTCCCTGAAGAATATACCCTCC
		R: CGCTATTGAGCACAGATACGAG
ROR-γT	NM_011281	F: GAACCAGAACAGGGTCCAGA
		R: CGTAGAAGGTCCTCCAGTCG
pIgR	NM_011082	F: ATGAGGCTCTACTTGTTCACGC
		R: CGCCTTCTATACTACTCACCTCC
***Housekeeping genes***		
TBP	NM_013684	F: CAAACCCAGAATTGTTCTCCTT
		R: ATGTGGTCTTCCTGAATCCCT
HPRT1	NM_013556	F: TGGATACAGGCCAGACTTTGTT
		R: CAGATTCAACTTGCGCTCATC
RPLPO	NM_007475	F: ACTGAGATTCGGGATATGCTGT
		R: TGCCTCTGGAGATTTTCGTG

### ELISA

IgA antibody levels in small intestinal tissue were measured by enzyme-linked immunosorbent assays (ELISA) essentially as described in Paerewijck *et al*.^8^. Total IgA and *Giardia*-specific IgA levels were measured in intestinal samples from infected and uninfected C57BL/6 mice at day 7, day 21 and day 60 post-infection. Small intestinal tissue was ground into powder in liquid nitrogen. A water-soluble protein extract was prepared by dissolving the powder in 150 mM PBS. After centrifugation at 16 000 rpm for 15 minutes, the supernatant was frozen at −80 °C until further use. Protein content of the supernatant was measured with the Pierce BCA protein assay (Thermo Scientific). For measurement of total IgA, Maxisorp 96-well plates (Nunc) were coated with 2 μg/ml sheep anti-mouse IgA (Sigma-Aldrich), for 16 h at 4 °C. For measurement of *Giardia*-specific IgA levels, plates were coated with *G*. *duodenalis* trophozoites. Hereto, trophozoites that were kept in axenic culture in modified TYI-S-33 medium, were centrifuged for 5 min at 800 g, re-dissolved and washed 3 times in PBS. Next, they were re-dissolved in 8% formaldehyde solution. Two mL of formaldehyde solution was added to trophozoites derived from 10 mL of culture. Hundred μl of this solution was added to each well of the plates, which were pre-incubated with poly-L-lysine (Sigma-Aldrich). Trophozoites were allowed to attach to the plates for 48 hours and the plates were subsequently dried at 37 °C for 24 hours. After washing with PBS-Tween20 (0,05%) solution (PBST), all plates were blocked with 2% bovine serum albumin (BSA) (Sigma Aldrich) in PBST. After blocking, the plates were incubated for 1 hour at room temperature with the intestinal protein extracts (200 μg/mL). For measurement of total and *Giardia*-specific IgA levels, the plates were washed and incubated for 1 hour at room temperature with goat anti-mouse IgA-HRP (Sigma-Aldrich) in a 1/1000 dilution in PBST. All reactions were visualised with ABTS in ABTS buffer (Roche). Optical density was measured at 405 nm with a Tecan plate reader (Tecan).

### Statistical analysis

Statistical analysis was carried out using GraphPad Prism software. For the A one-way ANOVA followed by a Dunn’s multiple comparison test was used to determine differences both within and between the control and infected groups. A P-value ≤ 0,05 was considered significant.

## Results

### Dynamics of a *G*. *muris* infection in pre-weaned mice

**Figure 2:**
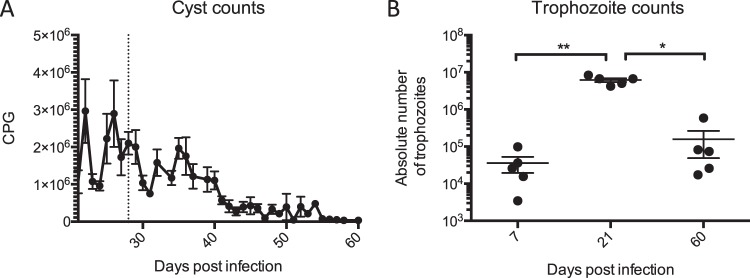
Dynamics of a *G*. *muris* infection in neonatally infected mice. (**A**) Cysts present in the faeces of infected mice were monitored every 2 days from day 21 p.i. until day 60 p.i., corresponding with day 25 and day 64 after birth respectively. Mean numbers of cysts per gram faeces obtained from 5 mice at every time point are depicted, with SEM as error bars. The time of weaning, i.e. 28 days after birth, is indicated by a dotted line. **(B)** The absolute numbers of trophozoites present in the small intestine obtained from 5 infected mice, each represented as one dot with SEM as error bars, are shown at 7, 21, and 60 days p.i. (*p ≤ 0.05, **p ≤ 0.01).

To overcome the technical challenge of collecting individual faecal samples in mice younger than 3 weeks, secretion of cysts in the faeces was only monitored from day 21 post infection (i.e. 25 days of age) onwards. The results of the cyst and trophozoite counts are shown in Fig. 2. While adult mice normally clear an infection with *G*. *muris* in approximately 3 weeks’ time^21^, cyst counts in the neonatally infected mice only started to consistently decrease from day 40 post infection onwards (Fig. 2A). Likewise, trophozoite counts in the neonatally infected mice significantly increased between day 7 and day 21 post infection (i.e. 11 and 25 days of age, respectively), after which they were significantly decreased at day 60 p.i. (Fig. 2B). No significant differences between male and females pups were observed.

### Kinetics of the intestinal immune response following a *G*. *muris* infection in pre-weaned mice

**Figure 3:**
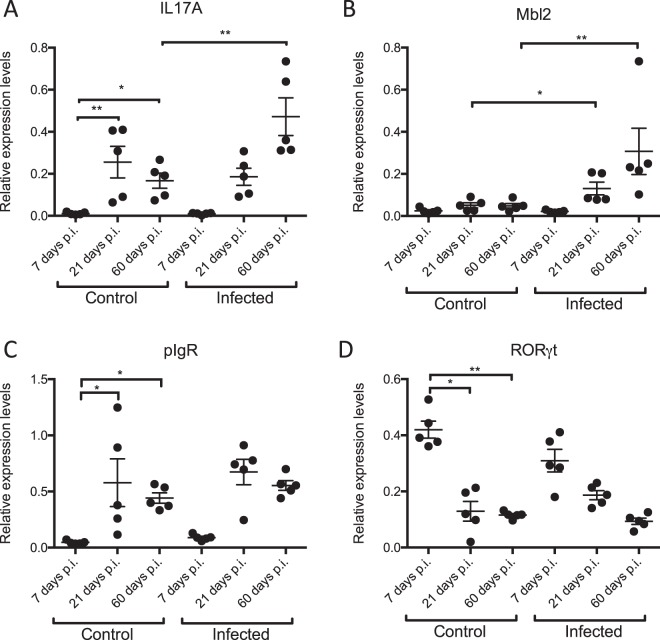
Kinetics of the intestinal immune response following a *G*. *muris* infection in neonatal mice. Relative mRNA expression levels of several immune-related genes were measured by qPCR at days 7, 21 and 60 p.i., both in infected and uninfected control mice. Every dot represents data obtained from 1 mouse, with SEM as error bars. (*p ≤ 0.05, **p ≤ 0.01).

The kinetics of the intestinal immune response in the pups was investigated by analysing transcript levels of a range of genes previously shown to be crucial in the protective immune response. These included the cytokine IL-17A, the RAR-related orphan nuclear receptor RORγt which promotes thymocyte differentiation into pro-inflammatory Th17 cells, mannose binding lectin 2 (MBL2) which is a pattern recognition receptor of the innate immune system and the polymeric immunoglobulin receptor (pIgR) which is needed to transport locally produced IgA across mucosal epithelia. The results of the quantitative PCR analyses are summarized in Fig. 3. The results obtained for the negative control mice indicated an age-related expression pattern for some genes. IL-17A transcript was barely detectable at an age of 11 days, but was significantly increased in 25 and 64 days old control pups (Fig. 3A). A similar expression pattern was observed for pIgR (Fig. 3C). Transcript levels of RORγt on the other hand significantly decreased when the pups got older (Fig. 3D). Finally, no significant changes were observed for Mbl2 (Fig. 3B). Comparison between the control and the infected pups showed a significant increase of IL-17A levels at day 60 post infection (i.e. 64 days old), whereas Mbl2 was significantly upregulated at days 21 and 60 post infection (i.e. 25 and 64 days old respectively) (Fig. 3A,B). The transcription patterns of pIgR and RORγt were not impacted by the *Giardia* infection.

**Figure 4:**
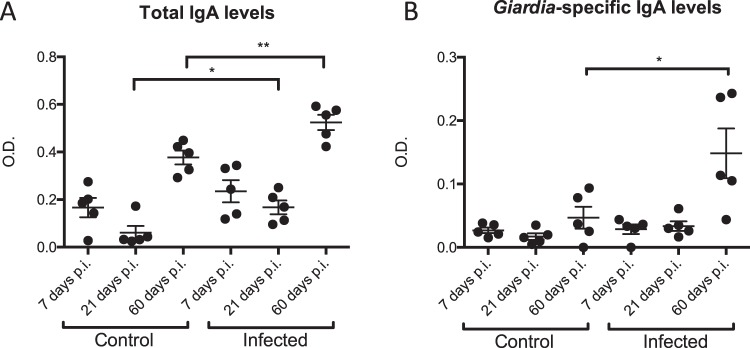
Intestinal IgA levels in uninfected control and *G*. *muris* infected neonatal mice. (**A)** Total IgA levels and **(B)** *Giardia*-specific IgA levels in the small intestinal tissue were measured by means of ELISA at days 7, 21 and 60 p.i. Optical density is shown in the graphs. Every dot represents data obtained from 1 mouse, with SEM as error bars. (*p ≤ 0.05, **p ≤ 0.01).

In addition to the transcript levels of the above mentioned genes, both the total amount of IgA’s and the levels of parasite specific IgA’s present in the small intestinal tissue were measured by ELISA in both control and infected pups. The results are shown in Fig. 4. In control mice, total intestinal IgA levels significantly increased over time (Fig. 4A). Furthermore, at days 21 and 60 post infection (i.e. 25 and 64 days old), there was a significant increase in total IgA levels in the intestines of infected mice compared to uninfected mice (Fig. 4A). In addition, a significant increase in *Giardia*-specific IgA’s was observed in the infected mice 60 days post infection (i.e. 64 days old) (Fig. 4B).

## Discussion

The outcome of this study shows that a *G*. *muris* infection in pre-weaned mice failed to trigger an IL-17A response, which could explain the prolonged course of infection in comparison to older mice. Only after weaning, a protective intestinal immune response started to develop, characterized by an upregulation of IL-17A and Mbl2 and the secretion of parasite-specific IgA. This combination of complement activation and parasite-specific IgA’s has previously been shown to be crucial to combat a *Giardia* infection^8,22,23^.

Neonates are generally more susceptible to intestinal infections than adults as their intestinal immune system is still in development following birth^24,25^. At the time of birth, the intestinal mucosal layer is typically thinner and there are less Paneth cells and immune cells present in the intestinal tissue in comparison with older individuals^26^. Although Peyer’s patches have already been formed in the murine small intestine at the time of birth, it is only after weaning that they fully mature with the development of germinal centres and activated lymphocytes^25^. These Peyer’s patches, and in particular the resident macrophages that are present, play an important role in the initial steps of raising an effective immune response following a *Giardia* infection^27–31^. In the current study we have shown that the basal IL-17A levels in the intestinal tissue of the neonatal mice were very low. Although the cellular source of IL-17A following a *Giardia* infection is not yet fully elucidated, at least CD4^+^ T cells in the intestinal lamina propria seem to play a role^5^. In the small intestine of neonatal mice, under conditions of homeostasis, CD4^+^ T cells have an immature phenotype with a reduced synthesis of IL-17A, whereas activated T cells in the lamina propria only seem to be present after weaning^32^. In the present study, IL-17A transcript levels also significantly increased with age, even in the absence of infection. In contrast, transcript levels of RORγt decreased over time. Although this transcription factor has shown to play a role in Th17 differentiation it is also essential for lymphoid organogenesis, in particular lymph nodes and Peyer’s patches, which could explain its early expression following birth.

In addition to T cells, the presence or absence of antigen presenting cells in the intestinal tract following birth is most likely an important contributing factor. Lantier *et al*.^33^ previously showed that the susceptibility of neonatal mice to the protozoan parasite *Cryptosporidium parvum* was linked to the low number of CD11c + CD103 + dendritic cells present in the gut. Since it was recently shown that the depletion of CD11c + dendritic cells in adult mice impaired the clearance of a *G*. *muris* infection^34^, it would be interesting to also investigate the role of CD11c + CD103 + dendritic cells in the course of a *Giardia* infection also in neonatal mice. Similarly as in mice, also in calves the number of T cells is significantly lower in newborn calves (1–5 weeks of age) when compared to weaned calves^35^ and the functional and structural maturation of the Peyer’s patches takes a few months^36,37^. Above insights, together with the observations made in mice, could explain why the upregulation of IL-17A following a *Giardia* infection in cattle only becomes detectable after weaning, as previously described^4,6^.

Following birth, the colonization of the gut by the microbiota is a crucial factor in the full maturation of the intestinal immune system, including the development of lymphoid tissue^38,39^. For instance, germ-free mice have smaller Peyer’s patches and mesenteric lymph nodes compared to conventional mice^40^. The bacterial colonization of the gut has also shown to stimulate the expression of the polymeric immunoglobulin receptor (pIgR)^41^. Also in the present study, an age-dependent upregulation of pIgR was observed in the control mice during a period that coincides with gut microbial colonization. As pIgR is responsible for the transcytosis of soluble dimeric IgA’s inside the intestine, this could also explain the appearance of parasite-specific IgA’s in the intestine following weaning. Interestingly, the administration of broad spectrum antibiotics renders adult mice susceptible to infection with *G*. *duodenalis*^42^, whereas such intervention is not necessary in mice younger than 21 days^43^. The absence of a fully mature intestinal immune system can explain the susceptibility of neonates, but the mechanisms behind the increased susceptibility of adult mice following antibiotic treatment is still not fully unravelled.

Although neonatal mice seem to be immunologically incapable of clearing a *Giardia* infection before weaning, ingesting milk from immune mothers was shown to have a protective effect against a *G*. *muris* infection^44^. Similar observations have also been made in both calves and children receiving mother’s milk that contains anti-*Giardia* antibodies^45–47^. The consumption of such immune milk however does not result in the induction of sustained immunity against this parasitic infection, which implies that the individuals become susceptible again following weaning or when milk consumption decreases^44^. Nevertheless, reducing *Giardia* infections in neonates is still important. Recent cohort studies in children have shown that early infections, i.e. in the first 6 months of life, were significantly associated with stunting at an age of 2 years^48,49^, even when the *Giardia* infection was not associated with diarrheal symptoms. It was suggested that this could be due to the disruption of epithelial cells by *Giardia*, however experimental evidence for this hypothesis is still missing. The murine infection model used in this study could be an interesting tool to further investigate the mechanisms behind the long-lasting effects of a neonatal *Giardi*a infection.
